# Implementation evaluation of a whole systems approach (WSA) to childhood overweight and obesity in local communities: findings from the Children and Families Pilot in Wales, UK

**DOI:** 10.1186/s12889-026-27489-9

**Published:** 2026-05-04

**Authors:** Anna Kolosowska, Rochelle Embling, Sophia Bird, Ilona Johnson, Julie Bishop, Sara Long

**Affiliations:** 1 Health Improvement Division, Health & Wellbeing, Public Health Wales, 2 Tyndall Street Cardiff, Cardiff, CF10 4BZ UK; 2 DECIPHer, Spark, Cardiff University, Maindy Road, Cardiff, CF24 4HQ UK

**Keywords:** Childhood obesity, Whole systems approach, Implementation evaluation, Community-based intervention, Systems change

## Abstract

**Background:**

Whole system approaches (WSAs) are increasingly adopted to improve public health practice and outcomes. This paper reports on the evaluation of the Children and Families Pilot (referred to throughout as PIPYN), which is a combined WSA and nested family-based intervention (FBI) that aims to reduce levels of childhood obesity (age 3–7). Piloted between 2021 and 2025 in three local communities in Wales, United Kingdom (UK), this evaluation aimed to examine implementation of the WSA and FBI, and understand perceived impacts of both components, including system change across pilot areas, in relation to childhood overweight and obesity.

**Methods:**

Using a qualitative study design, 19 semi-structured interviews were held with PIPYN programme development stakeholders at the national level and local stakeholders involved in implementation. A documentary analysis was used to summarise pilot development and to contextualise interview findings. Thematic analysis was used to identify key themes and explore perceived impacts of the pilot.

**Results:**

Four thematic areas are presented to summarise programme viewpoints relating to: 1) effective systems working for childhood overweight and obesity; 2) collaborative communities; 3) Family-Based Intervention (FBI) implementation and uptake; and 4) barriers and facilitators to programme delivery. Across themes, stakeholders perceived the WSA as an effective method to support a systemic shift across agencies to prevent childhood overweight and obesity. Key steps involved strengthening stakeholder partnerships, adapting approaches to support local systems, and addressing gaps in weight management services through the FBI. Stakeholders identified substantial challenges in monitoring and evaluation, with data incompleteness for the FBI (particularly for anthropometric data) and limited capability to assess systems-level impacts, imposing constraints on evaluation of programme implementation and effectiveness.

**Conclusions:**

There is a need for well-defined intervention and system boundaries to guide set-up and implementation of WSAs. Related to this, future efforts require a focus on the development and promotion of applied, practice-oriented guidance for implementing WSAs. Clear monitoring and evaluation metrics are needed that are practice-friendly and integrated from the outset of programme delivery.

**Supplementary Information:**

The online version contains supplementary material available at 10.1186/s12889-026-27489-9.

## Background

Globally, the combined prevalence of overweight and obesity in children has doubled over the last three decades [1]. Childhood overweight and obesity is associated with multiple long-term adverse health consequences, ranging from an altered immune system that can contribute to metabolic, cardiovascular, and autoimmune diseases and cancers, to the development of pulmonary, renal, musculoskeletal, and gastrointestinal complications [2]. Rising overweight and obesity in childhood is a major concern, with long-term health and wellbeing impacts that continue through to adulthood [3–6].

Since publication of the Foresight report on overweight and obesity [7] and the Marmot Review on social determinants of health [8], there is wide recognition that complex health challenges like obesity are driven by complex interactions between biological, behavioural, social, cultural, environmental and political factors. These different layers of influence, as presented in the Socio Ecological Model [9, 10], provide a framework for complex systems perspectives, which posit that different levels of influence and agents interact to result in patterns of behaviour that are non-linear, unpredictable, and often resistant to single-factor interventions [11, 12].

Whole systems approaches (WSAs) are designed to take account of behavioural and intervention complexity. Though definitions vary in the literature, WSAs can be broadly defined as approaches that aim to understand and intervene across interacting layers of influence. There is recognition that system behaviour and change emerge from dynamic, multi‑level interactions [13]. WSAs are commonly understood as comprising several core elements. For example, Stansfield, South and Mapplethorpe identified eleven elements of WSAs in practice, spanning ‘involving communities’, ‘strengthening capacity and capability’, ‘scaling practice’, and ‘sustaining outcomes’ [14]. This means that WSAs are often centred on iterative development of interventions, coordinated multi-agency action, and responsiveness to local context, features which implicitly align with core systems principles [15]. Therefore, small changes in practice are believed to produce disproportionately large or delayed effects on public health outcomes, through reinforcing or balancing feedback loops, threshold shifts and emergent patterns of behaviour.

WSAs have become increasingly central to the prevention of overweight and obesity [13, 16]. However, despite an abundance of systems frameworks and guidance [17, 18], there remains a need for evidence-based practice to shift interventions beyond the conventional, linear models of cause and effect [19–21]. Internationally, there have been pilots of WSAs to address overweight and obesity at the community level, for example, in the Netherlands [22, 23], Australia [24–26] and the UK [27–29]. While these initiatives contribute valuable experience, there is a significant gap in studies that implement and rigorously evaluate systems approaches in ways that reflect the complexity and diversity of different local contexts within nations, particularly those involving multi-agency governance, system mapping, development of system-change narratives, intervention development, and mechanisms for monitoring system change [22, 30]. Existing guidance describes these components but rarely examines how they function together as interacting parts of a dynamic system.

Wales is a small, westernised UK nation where health is devolved from central UK government. One in four children in Wales are affected by overweight or obesity by the time they start school at age 4-5 years [31, 32]. Though there is limited-service capacity, acceptability and uptake of child weight management support interventions [33], wider system efforts to prevent and reduce obesity across Wales include Welsh Government's *Healthy Weight: Healthy Wales strategy* [34], the All Wales Weight Management Pathway for children, young people and families [35], and the Whole Systems Approach to Healthy Weight Programme (WSAHW) [36]. These policies and programmes aim to support prevention, early identification and treatment, as well as build system capacity and partnerships to influence drivers of healthy weight. Funded by Welsh Government within this remit, Public Health Wales launched the Children and Families Pilot (CFP, or herein referred to as PIPYN) in 2021. The PIPYN programme, piloted in three local authority areas by on-the-ground practitioners, aims to deliver a local WSA and embedded family-based intervention (herein referred to as FBI) to support families to achieve a healthy weight for their children. In this study, the WSA refers to efforts to influence the wider local childhood obesity system, while the FBI, which is nested within the WSA and forms part of it, refers to a multi-level intervention delivered within that broader change process. Notably, PIPYN does not treat the nested FBI as a fixed entity delivered within a system, but as a change process embedded within the system‑oriented approach itself. This design enables a more rigorous evaluation of how system components interact across diverse local contexts, and contributes a novel, real‑world example of WSA implementation and intervention development within a different national system and across different local contexts.

Guidance on evaluating systems approaches emphasises the need to develop an understanding of: a) the pre-existing system; b) implementation context; c) mechanisms of impact; and d) unintended consequences [37–40]. Research aims were therefore centred around these factors. We carried out an implementation evaluation of PIPYN to:

1. Explore if and how the early years system had evolved in each pilot area to support systems working for healthy weight, and to understand how these system-level shifts informed the implementation of the PIPYN WSA;
2. Develop an understanding of how the FBI had been delivered in each area, generating evidence on implementing a multi-component intervention embedded within a complex adaptive system;
3. Explore perceived impacts of the overall programme (WSA and FBI) on behaviour change outcomes;
4. Identify and summarise perceived barriers and facilitators affecting the implementation of the whole‑system programme, encompassing both the WSA and embedded FBI intervention.

## Methods

### Overview of PIPYN

**Fig. 1:**
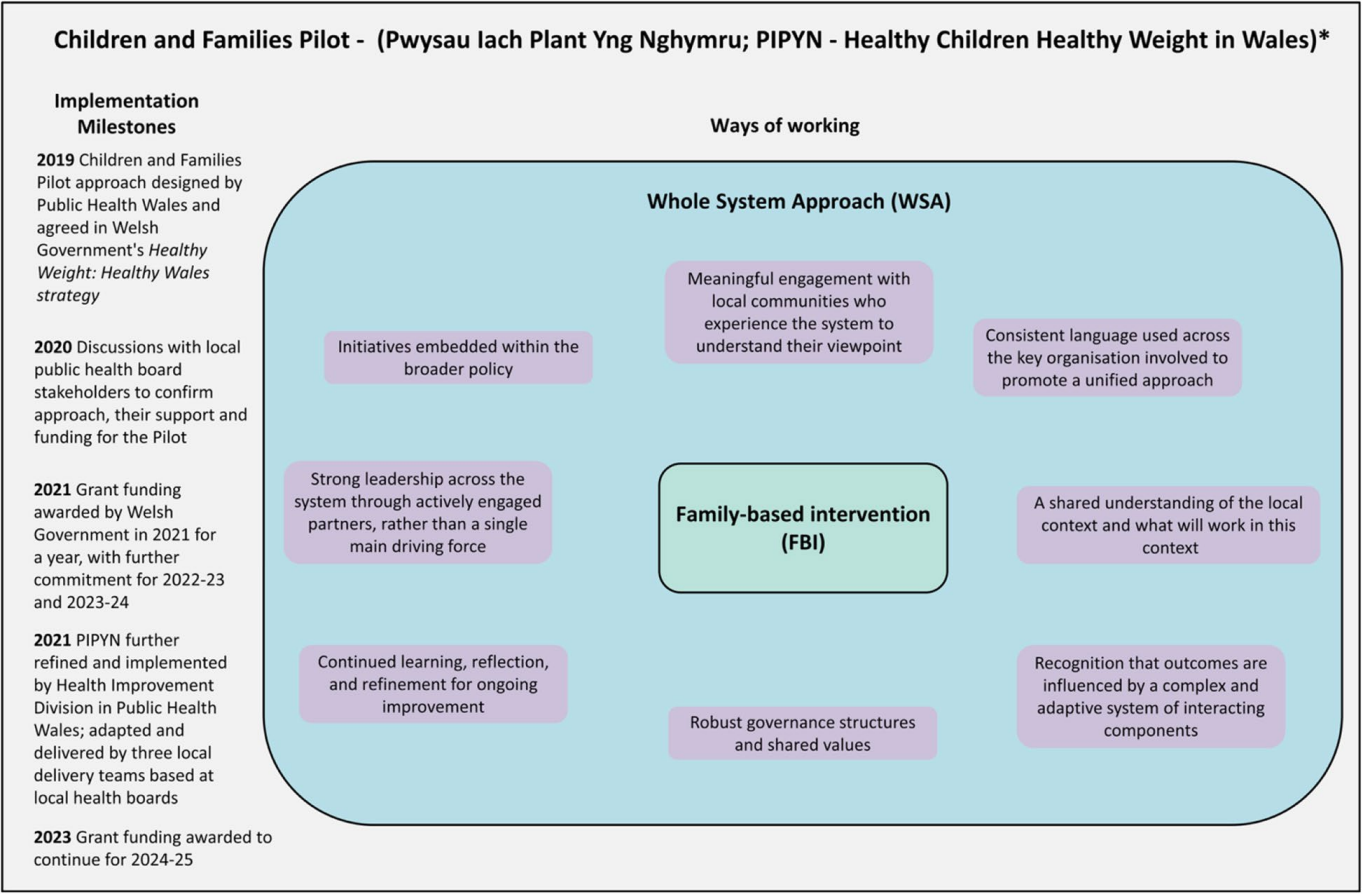
Overview of PIPYN programme structure and timeline. *Information presented in this figure was synthesised from a documentary review of programme materials and supplemented by published WSA guidance developed by Public Health Wales [41]

Figure 1 presents an overview of the PIPYN programme structure and timeline. PIPYN aims to change the local environment around families with children aged 3–7, while also providing direct family-level support through the embedded multilevel FBI. Designed and overseen by the national delivery team in the Health Improvement Division at Public Health Wales, implementation was piloted in three of 22 Welsh local authorities (LAs) representing local government administrative areas. Local delivery teams in each locality were responsible for delivery of PIPYN in its totality in their area, with oversight from the national delivery team. Local delivery teams consisted of professionals from either dietetic or project management backgrounds, and were locally managed by the health boards’ dietetics services. Pilot LAs were selected based on high levels of deprivation, overweight and obesity, and rurality. Further, one pilot area was selected based on an identified need for community services that better reach Black, Asian and Minority Ethnic populations. These characteristics were used to ensure the programme was tested across diverse contexts where the burden of childhood overweight and obesity, and the structural conditions shaping risk, were expected to be different. As this was a pilot programme, selecting areas with contrasting demographic and geographic profiles also offered opportunities for local areas to adapt and pilot the WSA and FBI components within their own contexts, while generating learning on feasibility and implementation across different community settings.

#### WSA component

The WSA component of the programme was designed by PHW using the definition set out by Bagnall et al. [13] and further informed by the Foresight Report [7]. These frameworks emphasised obesity as a dynamic, multifactorial public health challenge requiring coordinated action across sectors and levels of governance. The frameworks provided the systems science foundation from which local delivery teams could develop their WSA within their localities. The approach drew on ways of working integral to WSA practice, for example, establishing shared leadership structures, using consistent system wide language, engaging meaningfully with local communities, and grounding actions in the local context (see Fig. 1).

**Fig. 2:**
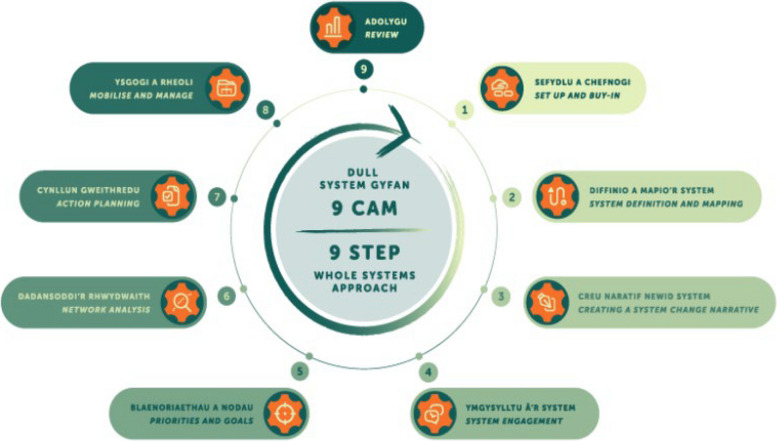
Nine Step Approach to Whole System Working in Wales (reproduced from Public Health Wales) [41]

Specifically, each pilot area was encouraged to adopt a WSA by defining and renegotiating its system boundaries, that is, by determining which actors, settings and determinants were most important for healthy weight in their area and population. They were also expected to work with national partners to understand the dynamic relationships shaping how each local system (i.e., local partnerships, services, governance arrangements and population needs) functioned and evolved over time, including how local partnerships developed, how processes and priorities shifted around the healthy weight agenda, and how emerging patterns of collaboration and influence supported intended outcomes of the programme. Activities included establishing multi-agency governance and reference groups; mapping the system to understand and define the local context; creating a system change narrative with local partners and organisations to identify shared priorities and goals (e.g., to build stakeholder relationships, ways of working, and the environment; Supplementary Material 1); and monitoring system change (i.e., changes in partnerships, delivery processes and local healthy weight activity). This was complemented by actions at the national level, which included: mapping of wider systems for overarching policies, strategies and interventions focussed on healthy eating and active families; network analysis in each area to understand relationships between stakeholders, causal relationships between influencing factors for healthy weight, and opportunities or leverage points for future action; and interviews and questionnaires with stakeholders and key organisations to obtain a baseline view of the system and system connections in each area. As the programme developed, the national development team created the ‘9 Steps to a Whole Systems Approach for Healthy Weight’ (Fig. 2.) [41]. This approach provides guidance on how to operationalise and implement a WSA.

Implementation of the systems component of PIPYN was further supported by external systems training sessions which included: access to online tools and guidelines, and an introduction to systems approaches, systems mapping, systems thinking, and systems evaluation. A Community of Practice (CoP) was also established to promote learning and development of the WSA. Each pilot area had a different pre-existing system, such as set up of services, partnerships, community assets and population needs (Supplementary Material 2), meaning that the introduction of the WSA and FBI took on a different focus and trajectory in terms of population focus, delivery model and success criteria as this was shaped by early years system structures, characteristics and population needs.

#### FBI component

For the FBI, families were eligible for professional or self-referral if they were motivated to change; had an eligible child aged 3–7 with a body mass index (BMI) around the 91 st percentile; and did not require other weight management support or have highly complex needs. The FBI was initially planned as a tailored one-to-one behaviour change intervention delivered by a Family Support Worker (FSW). The original intervention aimed to target the family food environment and diet (e.g., meal planning, shopping), family physical activity environment and opportunities (e.g., play, active travel), and wider supporting health behaviours (e.g., sleep, screentime). Together, these target core competencies of behaviour change which were underpinned by the COM-B Model [42]. The FBI offer and evaluation requirements were set in accordance with the All Wales Weight Management Pathway 2021 for children, young people and families [35] and National Institute for Health and Care Excellence (NICE) guidelines for the monitoring and evaluation of weight management services provision for children and young people [43].

Each area (Areas A, B and C) developed its FBI offer according to the local system context, with flexibility over delivery of core activities to enable local adaptation (Supplementary Material 3). This included a combination of individual and group-based family support, with programme dose and format varying across areas. Area A offered up to six weekly sessions delivered primarily in home settings with group support sessions also available, while Area B provided up to eight weekly sessions (1–1.5 h) delivered at home or community settings by the FSW. Area C implemented a settings-based model through schools and childcare services, combining a four-week digital offer with a 5-to-8-week face-to-face programme delivered by the FSW and Dietitians. Across all areas, delivery included topic-focused support on healthy eating, budgeting, cooking and family physical activity, alongside signposting to existing community programmes and services.

To support the evaluation of the pilot, each area was expected to collect monitoring and evaluation data for the FBI. This included a baseline goal-setting tool used at initial assessment, alongside questions around referral method, demographics and baseline child weight. A post-intervention assessment tool aimed to capture self-reported behaviour change and child weight at programme end.

### Study design

This evaluation consisted of two activities: semi-structured interviews with key stakeholders involved in the design, management and delivery of PIPYN; and a documentary review of programme development and delivery in each of the three pilot areas. Complex system frameworks and process evaluation guidance [38, 39, 44] were used to develop the interview schedule and an analysis framework for the documentary review. This included five key areas: identifying the context prior to implementation (e.g., what did the early system look like prior to PIPYN?), steps for implementation (e.g., what is implemented, and how?), mechanisms of impact (e.g., what are the intended impacts of the intervention, and how does the delivered intervention produce change?), influencing context (e.g., how does context affect implementation and outcomes?), and unintended impacts or consequences (e.g., what are the ripple effects of the intervention?).

Ethical approval was received from the Research Ethics Committee, School of Social Sciences, Cardiff University in October 2024 (ethics application number 678) and all activities were carried out in accordance with Cardiff University’s Research Integrity and Governance Code of Practice. Data collection and curation took place between October 2024 and January 2025.

### Data sources

#### Interviews

Semi-structured interviews (*N* = 19) were conducted with stakeholders to explore their perceptions and experiences of implementing the pilot. Across pilot areas, participants were identified and recruited using a combination of purposive and snowball sampling. Table 1 depicts characteristics of interviewees. Firstly, national stakeholders (n = 4) and local delivery team stakeholders (n = 8) were identified and interviewed, followed by interviews with broader stakeholders identified by the local and national delivery teams who were supporting the WSA and FBI (i.e., partners not directly involved in operational delivery, such as childcare and healthcare professionals in each area; n = 7). Stakeholders were eligible to take part if they were directly or indirectly involved in PIPYN planning or delivery.

**Table 1:** Stakeholder interviewees

Level	Role	N
National	Senior responsible officer	1
	Consultant lead	1
	Programme lead	1
	Programme co-ordinator	1
Local	Project co-ordinator	3
	Dietetic lead	2
	Family support worker	3
	Health visiting service	2
	Play worker/lead	3
	Community lead	1
	Primary school representative	1

Interviews were conducted online and recorded using Microsoft Teams (https://teams.microsoft.com/), lasting on average 40 min (ranging from 20 to 58 min), and followed a semi-structured interview guide (see Supplementary Material 4). All interviews were carried out by or in the presence of the same member of the evaluation team (AK). All participants were provided with a participant information sheet about the study and signed a written consent form prior to taking part.

#### Documentary review

To provide additional context for interviews, a documentary review identified and analysed programme and implementation documents (n = 31). Documents were purposively selected to capture different stages of programme development, from initiation to the most recent implementation period, and to provide insight into both processes and reported outcomes across pilot areas. This included programme initiation documents; governance and implementation guides; quarterly and annual progress reports; system maps; and advisory group meeting notes and transcripts. The documentary review was structured to capture pre-existing system, family, and community contexts; planned and implemented programme components, including delivery of the FBI and WSA activities; reported changes to local systems and family and community support; and adaptations over time, including barriers, facilitators and monitoring processes. Findings from the documentary review also informed development of the interview guide and helped contextualise timelines of programme development across areas. Data collection was supported by the national delivery team (Public Health Wales), who helped identify and facilitate access to documents.

### Data analysis

Interview recordings were transcribed via Microsoft Teams’ record and transcribe function and were reviewed and corrected prior to the analysis. Interview data was coded (AK) in NVivo 14 software (https://lumivero.com/products/nvivo/) and followed Braun and Clarke’s six step approach to thematic analysis [45]. As the initial and secondary codes were developed, weekly meetings were held between AK, SL, and RE to guide the emergent analytical structure and support theme development, and SB helped further contextualise findings. Initial coding took a bottom-up approach to capture experiences and perceptions of PIPYN among the stakeholders. This was followed by a top-down approach where coding and theme development were guided by established frameworks for the process evaluation of complex interventions and systems-level evaluation studies [38, 39]. In contrast to more traditional implementation evaluations where the intervention fidelity, reach and dose tend to be the primary focus [46, 47], this approach meant that themes extended beyond participant descriptions of discrete activities, thus being reflective of how governance structures, local delivery processes, and the diverse conditions across programme areas shaped stakeholder experiences and programme evolution over time.

Documentary analysis was conducted by two researchers (RE & AK) and followed a four-step READ approach of: reading the documents; extracting the data; analysing the data; and distilling the findings [48]. In line with the complex systems and process evaluation frameworks applied in the thematic analysis, a bespoke data extraction template was used to systematically capture and synthesise reported developments for each pilot area. As such, while coding of interview data remained inductive, extracted data from the documentary review were used to provide contextual and structural insight to enrich the top-down interpretation of themes. As the significance of themes stems from their conceptual relevance rather than the number of participants expressing them [49], documentary data allowed us to situate participant accounts within the broader programme context, identifying tensions, contradictions and misalignments that contributed to final themes. It also allowed us to interpret why some stakeholder accounts appeared conflicting, particularly between national and local levels, by linking these differences to documented variations in responsibilities, delivery timelines and implementation expectations as part of the overall narrative.

## Results

**Fig. 3:**
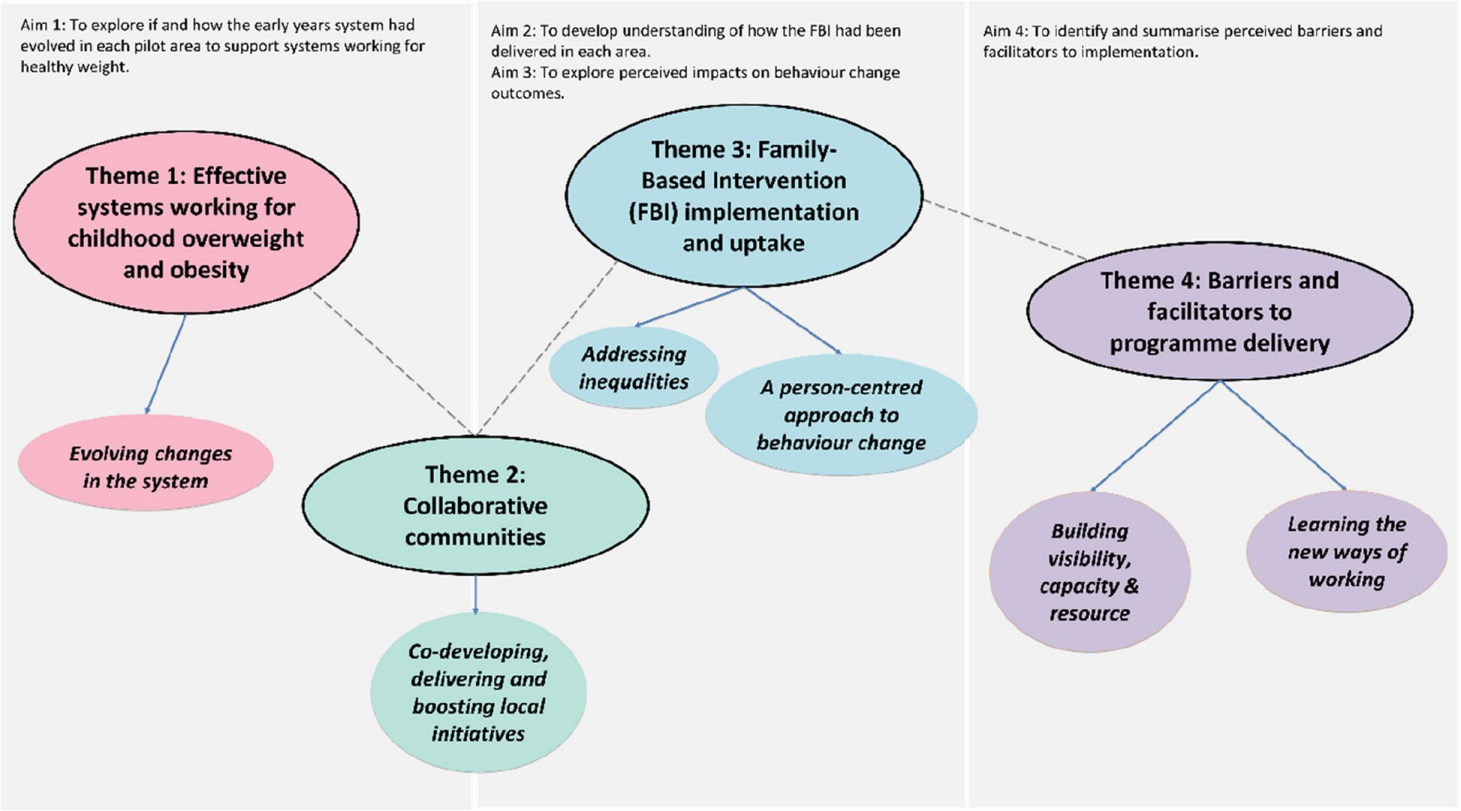
Thematic map showing the key themes and subthemes, as they relate to the aims of the study.

Four overarching themes were identified from interviews, relating to: effective systems working for childhood overweight and obesity; collaborative communities; Family-Based Intervention (FBI) implementation and uptake; and barriers and facilitators to programme delivery (Fig. 3). Each theme is presented below with illustrative participant quotes and is supplemented by findings from the documentary review, which helped to contextualise the overall narrative (see Supplementary Materials 1–3 for further detail).

### Theme 1: Effective systems working for childhood overweight and obesity

Documents and interviews with the development team supported PIPYN as a programme that was conceived as comprising of concurrent WSA and nested FBI activity. However, the FBI component was initially perceived by local delivery teams as the most important focus. The FBI was described as being more familiar and easier to set up than the WSA, which interviewees appeared to understand and value more fully in the later stages of the pilot. Many reflected that, in retrospect, spending more time developing the WSA would have helped to prepare the local landscape for implementation of the FBI. This meant that stakeholders often discussed perceiving the WSA and FBI as separate components, despite often recognising that the FBI was developed, adapted and delivered inside the wider system‑oriented process.

*“Because what was seen was, this is about a direct intervention, not the wider system narrative. So, in the early phases, all of the energy went into the nested intervention. And in fact, in some cases, you know, some of the teams [were] sort of saying, well, what systems piece, you know, they completely lost the wider system development component of the work.” Interviewee 14, National Stakeholder*

The systems component was universally recognised by interviewees as being crucial in preventing and addressing childhood overweight and obesity. Interviewees widely acknowledged the importance of developing shared language, collective goals, and shared assets across the local delivery system, including staff capacity, services, venues and partner networks. PIPYN was seen as the ‘glue’ or ‘central cog’ driving the healthy weight agenda and a way of fostering long-term environmental and cultural change. Local delivery teams were described as conduits for creating a shared vision, helping stakeholders to evaluate their efforts to promote healthy, accessible environments that support achievable healthy lifestyles for local families.

*“PIPYN on its own can't do any of this. It can support families in the short term and might have good initial outcomes, but long term we're almost setting the families up to fail if we're only doing intervention work. So for me, it's about having that shared ownership. It's about having that partnership structure. It's about improving accessibility and affordability to healthy lifestyle behaviours. …[It’s] partners recognising that and integrating into their service delivery.” Interviewee 11, PIPYN local delivery team*

*“We have been absolutely instrumental in being that centre point in a sense in the system, in enabling the conversations to take place between partners and organisations that maybe wouldn't have previously.” Interviewee 9, PIPYN local delivery team*

*“So it's a lifestyle change for the communities rather than individuals, because if we're all singing off the same hymn book and we're all given the same messages, it becomes a community information, which obviously when everyone's doing the same or similar thing then you [get] a healthier lifestyle with that.” Interviewee 18, Childcare sector stakeholder*

#### Subtheme: Evolving changes in the system

Stakeholders reflected positively on perceived impacts of the programme as a whole on their local systems. Interviewees across all three areas described creating new opportunities for local families as a result of the WSA, from encouraging healthy eating initiatives to providing cost free leisure activities. For example, through conversations with a local partner, one area secured free tennis sessions for families during the summer holidays. The programme's visibility facilitated conversations around children’s weight, as through its FBI offer, PIPYN provided a new contact point and local option for an offer of support to families.

*“So, we've partnered up with loads of sports providers, but we've also partnered up with the local Tennis Club …parents and children can access the Tennis Club for free.” Interviewee 2, PIPYN local delivery team*

*“…because once I’d opened that conversation, I had nowhere to go with it. You know, I could offer very little, I suppose, in terms of, you know, hands-on support. But now we've got PIPYN, it's allowed us to open those conversations and say, look, you know, there's no judgement here. There's no right or wrong. But have you considered XYZ? And this can be facilitated as a family. You know you get to spend really good quality family time, you know, improving the health and well-being of your family while spending you know that quality time, it's a really good initiative.” Interviewee 6, Local health board stakeholder*

While interviewees perceived positive impacts of PIPYN overall, some participants noted that long-term multi-sector action was needed to achieve cultural change, including normalising healthier food environments and sharing responsibility for healthy weight across sectors working with local communities. They highlighted that changes in childhood overweight and obesity rates are long-term outcomes, emphasising the importance of sustained, long-term investment in programmes like PIPYN to facilitate broader systemic change.

*“There's actually additional funding being given to one area because it's got that visibility and they see it as a way forwards and people are engaging with it. So, what you're seeing is momentum in the system.” Interviewee 4, National Stakeholder*

*“We're not going to see that trend of that child suddenly becoming of a healthy weight, that's going to take time, and that sustainability needs to stay. But hopefully, you know, giving PIPYN the chance to stay and keep flourishing and growing.” Interviewee 19, Local health board stakeholder*

### Theme 2: Collaborative communities

Facilitating community stakeholder collaborations to drive the healthy weight agenda was perceived to be the greatest success of the programme among interviewees. Many felt that collaboration would not have occurred without the PIPYN teams facilitating the WSA, as they successfully engaged a broader range of stakeholders such as third-sector organisations. For example, one area delivered training for food bank staff to improve the nutritional value of the food parcels offered. Interviewees reported that strengthening local partnerships and building network relationships was a core aspect of the systems approach.

*“I think we filled a gap where …by bringing people together, we've been able to add value to all of them…we've been able to bring them together. All right? We can work together. You know, it doesn't eat into our profit. It doesn't eat into your profit. We can share resources. You can share personnel. We can do it.” Interviewee 9, PIPYN local delivery team*

*“And then collaborating with them with other agencies and groups to bring in (…) They've told us what they want and how they want, where they want it. Let's make it happen and then if we can't make it happen, we will know somebody that that does or will want to support. So, I think that's where this with everyone speaking the same language with everyone kind of aiming for the same kind of goals and objectives.” Interviewee 5, Local leisure sector stakeholder*

Action from across the system (e.g., local services, community settings and strategic organisations) was perceived to be necessary for the WSA; from local community (bottom-up) to local authority-wide organisational levels ('top-down'). The interviewees talked about effectively driving change in immediate community settings (e.g., playgroups, childcare), as well as achieving county or organisational-level change impacting local policy (e.g., school meal provisions, food environment). The latter was sometimes perceived as being beyond the scope of local practitioners, as change was believed to require 'top-down' intervention. There were also perceived challenges of cross-organisational and professional collaboration, particularly concerning their ability to influence the strategic local context within their local authority or health board.

*“…What happens with systems working is everybody drops the level or two. [county level vs local community] So they always go into individual interventions rather than saying, well, why are we, you know, always educating people or trying to tell people to do things differently when it's actually quite hard for those families.” Interviewee 4, National stakeholder*

*“…Trying to access the GP cluster meetings. I've countlessly emailed in order to get in there to discuss with GPs about the importance. They're all aware of the agenda of the 10-year programme to reduce childhood obesity and yet we're not being able to get into the cluster meeting.” Interviewee 19, Local health board stakeholder*

#### Subtheme: Co-developing, delivering and boosting local initiatives

Across all pilot areas, interviewees reflected on the benefits of PIPYN and how it could enhance, extend or boost local initiatives and activities, particularly as this related to the WSA. The programme also enabled access to Continuing Professional Development (CPD) offers around health improvement, enhancing capacity to support the healthy weight agenda within local systems.

*“... linking in with one of our partners …they were starting a new project as around that access to fruit and vegetables. So they were able to use the learning from that report to develop the project that they were doing. So from that they've developed this planet card which is you know linking in with some of the local markets and stuff …looking at ways that we can make more culturally appropriate food more affordable, but then also looking at sustainability as well.” Interviewee 16, PIPYN local delivery team*

Specifically, interviewees talked about PIPYN delivery teams initially fostering stronger relationships with schools, statutory childcare (e.g., Flying Start, a Welsh Government programme supporting families through funded part-time childcare, enhanced health visiting, parenting support, and speech, language and communication [50]), school nursing, Health Visiting, and leisure services. Over time, this expanded to include wider system partners such as the third sector, with food poverty partnerships and ethnic minority support organisations highlighted as key examples. Interviewees reported that this led to more coordinated action, where services and interventions (including the FBI) complemented each other, alongside additional networking and training opportunities supported as part of the WSA.

*“And actually in practice it's been really good. They've been able to complement each other so the [physical activity] side have opened up doors for [PIPYN] to do some more activity and open up like you know, access to leisure centres and family fun days. And then [PIPYN’s] been able to support them with, like, nutrition courses and nutrition advice and stuff. So yeah, there it was. It was really like music to my ears.” Interviewee 12, National stakeholder*

*“It's an endless [stakeholder] list, really, because [we're] forever evolving and changing. So that list, I think, is an endless list. You know, today we could be asking schools. Tomorrow we'll [ask] Flying Start.” Interviewee 15, PIPYN local delivery team*

PIPYN was perceived to be filling a gap within a local support and referral system for families and frontline professionals. For example, Health Visitors, school nurses, and Flying Start providers sought nutrition and physical activity guidance from PIPYN teams more broadly, in addition to recognising the value of the FBI for families in the early years.

*“Or even, you know, prenatal, neo-natal and family. So there's a lot of scope there. …Even sort of pregnancy being part of, I don't know, maybe some parenting programmes. I don't know how strong the links are with parents, with parenting and PIPYN. Maybe an area that could be strengthened.” Interviewee 3, Childcare sector stakeholder*

### Theme 3: Family-Based Intervention (FBI) implementation and uptake

The embedded FBI was perceived by interviewees to address gaps in child weight management services, offering an adaptable, person-centred, intervention that addressed inequalities (e.g., for those affected by deprivation or rurality, and by increasing access for specific communities). Despite its perceived acceptability over traditional dietetics provision, interviewees noted that stigma around child overweight and obesity continued to affect family engagement. Perceived challenges around child BMI data collection, such as perceptions of stigma, led some areas to opt out of monitoring child weight outcomes, impacting the ability to evaluate programme impacts on BMI over time.

*“I think the second one for me is about how we have the conversation about weight in a way that gives people the confidence to participate and to understand that it's not their fault. And I don't think we've cracked that one yet. So, what we've got is the fact that people will actually really not have the conversation at all. So, there's an avoidance of having the conversation’” Interviewee 14, National stakeholder*

*“But we fed back quite quickly that actually measuring children and …saying they can only access the service if they're over the 91 st centile was going to be quite detrimental in our communities. We've done a lot of research around what would help them engage, what would be a barrier and that mention of weight, that weighing, that access criteria, was always going to be a challenge for us. And when some of the other criteria said, well, the index child needs to be over the 91 st centile or evidence of familial obesity or, you know, and a few other things I was like, well, familial obesity for us, that's a population health issue across [the local health board].” Interviewee 11, PIPYN local delivery team*

Interviewees described how, as intended, FBI offers varied across pilot areas as local teams adapted to local population needs and values, reflecting their efforts to define and work within system boundaries as part of the WSA. While initially planned for at home one-to-one delivery, settings and modes of engagement differed. Informed by community consultations, some areas opted for group sessions, leading to higher family uptake and engagement. Transport and venue issues in other areas required flexible, combined delivery approaches to support inclusion (e.g., online sessions).

*“Generally, [sessions were] held in schools or the local community centre. It’s really good for those people that are not able to travel or haven't got access to public transport, things like that. So yeah. Definitely schools. Because it just makes, it just makes it easier to access. You know, a wider audience, and it's easier for parents. They're there anyway.” Interviewee 6, Local health board stakeholder*

Interviewees reflected that in the context of recovery after COVID-19 restrictions, FBI delivery faced low referral rates, particularly from GPs and Health Visitors. Referral criteria were a key barrier to enrolment, including an inability to identify eligible children, for example, due to the weight of 2- to 3-year-olds not always being collected during Health Visitor appointments, and children being referred with BMIs outside of the eligible range and narrow age criteria. This prompted suggestions to expand the offer to younger and older children, illustrating how pre‑existing system structures and dynamics (e.g., workforce pressures, disrupted service pathways, and shifting organisational priorities) shaped the implementation of the nested intervention within the WSA.

*“In the initial phases, it was kind of we were at the mercy of the health visiting teams, so that was initially how it was all set up […] in such a way that we were to receive referrals for families from visiting health personnel who were doing the 27-month checks and weights and things like that. They didn't materialise, still not materialising, and trying to understand why health visitors are not referring in when we know that the numbers are there.” Interviewee 9, PIPYN local delivery team*

#### Subtheme: A person-centred approach to behaviour change

The FBI was designed to be a behaviourally informed, person-centred offer with goal-oriented exercises to achieve behaviour change. Interviewees talked about how delivery teams worked with families to set realistic and achievable goals that took account of psychosocial dynamics and potential stigmas in the local system context. Wider stakeholders from local health boards and community settings consistently praised the teams as passionate, non-judgmental, friendly, and approachable, with sessions being both informative and engaging. This approach was considered by interviewees as crucial for enabling lifestyle changes among families.

*“It's very clever, the way they do it, because from speaking to families, […] they don't feel pressured or judged by the team. They feel, you know, empowered when they've completed that course” Interviewee 6, Local health board stakeholder*

*“I think because of the way the programme is run, it's not so regimented, it's interactive. You're getting in there cooking. You're giving examples of different physical activity. […] I think it's really powerful. […] if in long term that can affect the statistics, then you're onto a winner”. Interviewee 17, School sector stakeholder*

#### Subtheme: Addressing inequalities

Addressing inequalities was seen by interviewees as a hallmark of the FBI offer, enabling a focus on disadvantaged communities. A targeted approach was perceived to facilitate adaptation to the needs of those affected by deprivation, poverty, and rurality, which was key to integrating and adapting to learnings from the WSA. Interviewees in some areas perceived challenges linked to disadvantage, for example, the need for additional support around self-feeding and fine motor skills. Another area addressed cultural and religious barriers to accessing support and activities (e.g., lack of single-gender events or spaces), providing culturally appropriate and translated materials and ensuring access to culturally relevant fruits and vegetables.

*“And it's not just around food and activity, it's everywhere, it’s homework, it’s tying shoelaces, it's learning to use a knife and fork. And it's like, OK, so for us and the difficulty is OK, can we positively impact this? Do we have the power, is it our remit to positively impact and say to parents …well, it's parenting at the bone you know. Are you taking responsibility for growing your healthy child?” Interviewee 9, PIPYN local delivery team*

### Theme 4: Barriers and facilitators to programme delivery

Interviewees across all pilot areas discussed implementation barriers and facilitators. Branding, visibility, and cross-organisational collaboration was perceived as supporting PIPYN's overall success. Key challenges included 'learning as you deliver,' evaluation activities, navigating new partnerships, and the uncertainties of funding and short-term contracts.

#### Subtheme: Building visibility, capacity & resource

Documents showed that a branding strategy around the name ‘PIPYN’ (Pwysau Iach Plant Yng Nghymru; PIPYN—Healthy Children Healthy Weight in Wales) was developed to facilitate engagement with local stakeholders and families. Interviewees acknowledged that as the pilot progressed, PIPYN gained momentum, becoming a well-recognised and trusted resource in each area. This related to both the FBI (e.g., in terms of referrals and uptake), as well as wider activities arranged as part of the WSA (e.g., community engagement events, partnerships, and cross‑sector initiatives developed through the system‑mapping and governance structures).

*“So rather than me just saying the project. I'd say probably the foundation and draw of that is that trusted resource that we've [got]. That being in the Community, that brand …Building that reputation that you know, we've listened to the community, we've put things in place, we work with the community.” Interviewee 7, PIPYN local delivery team*

*“Because obviously when we go out and about into [area]. We’re known by our first names. Now we're known as PIPYN. …..And yeah, wherever you go, really, you hear people saying, ‘Oh, yeah, PIPYN does this, PIPYN does that.’ Or whenever you go to a school, they've all got, like, display boards of PIPYN up.” Interviewee 2, PIPYN local delivery team*

An engagement strategy employing social media was seen by interviewees as being crucial for raising community interest and awareness (e.g., via local groups) and increasing referrals for the FBI. One area enhanced visibility through social media posts and videos, tagging local partners to increase reach and engagement. Interviewees also recognised the importance of engaging local families to build brand familiarity and rapport as part of wider WSA activities that aimed to strengthen connections across the local system and improve access points into the FBI. For example, delivering activities in informal settings was perceived to increase trust and was viewed as a mechanism for enabling teams to listen to the community, understand their needs, and identify barriers that the programme as a whole could address, strengthening pathways between system actors and ensuring programme delivery was responsive to local contexts and system dynamics.

*“You know, I think within a year or something, it was like everybody had heard of PIPYN. Most people had met PIPYN or knew something. So I think yeah. And their social media. I think, you know, they kind of hit the road running, didn't they when they first came in? So I would say the success is meeting with all the key partners, agencies, I'm really, you know, getting all those schools on board.” Interviewee 3, Childcare sector stakeholder*

While PIPYN was delivered or overseen by the local dietetic teams, some interviewees recognised the importance of the role of public health in steering brand messaging. This was deemed by interviewees as crucial to expand the programme's remit beyond nutrition and dietetics and ultimately to steer the WSA, reinforcing the need for the WSA and nested FBI to interact as part of a single, coherent system‑level programme. Consequently, local public health teams were identified as key partners for facilitating a collaborative approach.

*“But I think just having that stronger public health leadership for the programme in the area. Because even though I think the dietitians …you know they brought their own skill set, I feel like it ends up being quite focused on the nutrition element. I don't mean to be disrespectful by that, but it did. Whereas I think if you've got a what, somebody you know who's got that public health kind of wider thinking with them, it will naturally, you know, ensure that wider system doesn't get lost. And that's the bit we're really focusing on.” Interviewee 13, National stakeholder*

Interviewees noted that pilot areas with existing dietetics weight management services utilised PIPYN funding to extend the existing offer. Wider partner collaboration was seen as crucial for creating health-promoting activities and community-based provision that could continue to support families beyond the FBI sessions. However, relating to system-level constraints, such collaboration was often constrained by broader system pressures, including limited funding and short-term delivery timeframes. Additionally, interviewees reflected that local teams on short-term contracts, often not embedded in other services, faced challenges with workforce retention. Funding uncertainties were reported as a primary barrier to the programme future, highlighting the need for 'top-down' or national-level support to build necessary PIPYN delivery infrastructure (e.g., via local health board or policy change). As such, interdependencies between workforce capacity, governance and funding all shaped how the WSA and its nested FBI were implemented and sustained across the local systems.

*“I've been around long enough to see there's some fantastic schemes and initiatives, but they don't get the funding and the supports that should be there. I think some of the people, the powers that be, sometimes are too quick to move on to the next thing when really, they've already struck gold with something that's clearly having an impact.” Interviewee 5, local leisure sector stakeholder*

#### Subtheme: Learning the new ways of working

All interviewees acknowledged that PIPYN involved 'learning as you deliver,' with the programme evolving during implementation and the national and local delivery teams learning about WSAs and how to implement WSA components as they happened. Interviewees from the national team highlighted initial delays in set-up and delivery due to resource pressures as the programme was launched around the COVID-19 pandemic. This led to unplanned delays in provision of WSA guidance to local teams and explained some early uncertainties around delivering the programme. Interviewees also reflected on navigating new partnerships and unexpected outcomes, adapting delivery to better engage families as part of the FBI despite referral barriers.

*“We had a very short time scale to implement it. And that was one of the most difficult things. So we had to try and set up in the three different areas... The intervention side of it, plus the whole system stuff at the same time. …I think if you took a step back, you would probably in hindsight …do the whole system approach and then you would do the intervention. We were doing it at the same time, so we were trying to manage both these things. Our role within that was to manage and kind of oversee the local areas through that process [of] setting up, implementing it, taking it forward and guiding them through that and taking them on the journey with us. …But that probably wasn't as slick as it could have been due to capacity mainly I'd say, on both ends.” Interviewee 13, National stakeholder*

*“We've done some of the mapping work and some of the consultation. Thinking about all those other tools that you can use as well, [that] probably wasn't until later in 2023 where we started more kind of focusing on that more. And then we had those workshops. Then at the beginning, the beginning of this year, to kind of do and kind of produce that causal map. (…) It probably wasn't until a good year and a half into the project before we started to think we need to work a little bit differently.” Interviewee 16, PIPYN local delivery team*

Interviewees from local teams often described the challenges of learning about systems approaches, especially where stakeholders were unfamiliar with systems thinking, tools, and language. Despite access to training, WSA learning sessions were perceived as overly academic, missing opportunities to guide practical application, for example, via case studies. Interviewees acknowledged that they were still developing their understanding of systems working, requiring more time to integrate this into PIPYN locally particularly as part of the FBI. They also recognised the importance of allowing time to share this learning with expanding networks, fostering buy-in for consistent language and priorities.

*“I think when we started doing it, it was new to us all to get into that style of thinking, which is why we then asked [University] to do us a few sessions to get us into that kind of way of thinking around it really. So we're kind of learning and bringing people along with [us] at the same time. But I think it's just one of those things. I don't think it is a straight line, it's something that you can apply parts of it to. ….” Interviewee 13, National stakeholder*

Interviewees perceived monitoring and evaluation to be challenging. Despite provision of co-developed templates at the beginning of implementation in documents (2021), questions around the purpose and acceptability of including certain measures (e.g., BMI) led to incomplete data particularly for the FBI. Consequently, as noted by some interviewees, exploring impact on overweight and obesity within the pilot evaluation timeframe was not possible. Interviewees also described a need to better capture impacts of systems work as part of the WSA. Using systems software (e.g., Kumu for systems mapping) was deemed difficult, and early evaluation was perceived as challenging due to limited understanding of systems working among local teams and partners. Interviewees from the local delivery teams often noted the challenges of evaluation and monitoring tools that were difficult to use with the families accessing the FBI.

*“The way it's been intended wasn't supposed to be highly onerous, but for some reason it's felt onerous and I'm not sure why. And I think there are some challenges in demonstrating the longer-term as we know that people are saying they are making changes.” Interviewee 4, PIPYN national delivery team*

*“I've had meetings, and I've just been constantly feeding back and saying, can we really sit down together and look at these and talk? Thrash it out. It's not just the process and the questions. It's both. Especially when we're using interpreters, families don't understand. I mean, I don't even understand some of the questions and I know what we're looking for. You know, when I don't get the way some of the questions are worded. Plus we're using interpreters and things like that. And families are like, I don't even know what that means. We're not capturing it all properly.” Interviewee 7, PIPYN local delivery team.*

## Discussion

This study evaluated how a combined WSA with a nested FBI, designed to improve levels of childhood obesity (age 3–7), was operationalised, implemented, and experienced across three pilot areas in Wales, UK. The evaluation aimed to explore how the local early years system had evolved in each PIPYN pilot area, to understand implementation and early perceived impacts of PIPYN, and to identify potential barriers and facilitators for implementation. Findings highlighted interviewees valued a locally implemented WSA. Across stakeholder groups, the programme was perceived to have created early change within the local delivery system by strengthening relationships across services and organisations, building a shared agenda for healthy weight, and improving the visibility and recognition of local preventive efforts in the community. With its tailored approach, the FBI was perceived to address gaps in child weight management services, offering an adaptable, person-centred intervention that addressed inequalities in deprived communities.

As a result of the WSA component of PIPYN, new partnership structures were perceived to have strengthened capacity across services and organisations, enabling pilot areas to trial a range of community-based initiatives on healthy food, physical activity and parenting support. The design and delivery of the FBI added further capacity by establishing a tailored early years weight management offer. Collectively, these developments show how locally embedded WSAs can align action across the local early years services and partnership system, consistent with frameworks that emphasise cross-sector collaboration and shared ownership [18, 20]. PIPYN was repeatedly described as a mechanism that brought sectors together, supported shared priorities, and improved coordination across childcare, schools, health visiting, leisure services and the third sector. Overall, the pilot strengthened the relational infrastructure and collaborative practice that is needed for long-term system change [20]. Stakeholders also expressed belief in the value of WSAs and commitment to their principles, both factors previously identified as critical for progress in place-based system change efforts [27, 28, 51].

WSAs are promoted as effective approaches for improving health outcomes [13, 17, 18]. Yet there remains limited practical guidance on how to implement them from the outset, a persistent gap in the literature that was reflected in PIPYN set-up and implementation [17, 19]. In the early stages of implementation, local stakeholders described uncertainty about how to operationalise a WSA. Interviewees at the national level acknowledged delays in programme set-up, driven by resource pressures and difficulties providing clear, practice-oriented guidance. As a result, local teams were required to develop systems thinking skills, build partnerships and deliver interventions at the same time, often without shared frameworks or tools. This led to an initial focus on the more familiar FBI component, which was perceived as easier to implement than broader systems work. Outputs from PIPYN, such as the ‘9 Steps to a Whole Systems Approach for Healthy Weight’[41], and the adoption of a more gradual support pathway resembling a stepped-care model (i.e., support delivered at increasing levels of intensity according to need), provide concrete examples of how WSAs and embedded support offers such as the FBI can be structured, sequenced and sustained.

Study findings highlight the need for multi-level stakeholder action [9, 10] , as local practitioners cannot achieve whole system change alone. In this study, local practitioners often felt they lacked the power to effect broader change, making support from higher‑level and more established structures, including local public health teams, essential for building system capacity and influence [28].

### Implications for policy and practice

This study highlights a central implementation challenge: that WSAs are difficult to evaluate without clear, practice‑friendly monitoring and evaluation frameworks. This challenge leads to gaps in evidence around mechanisms of change, system dynamics and impacts of WSAs [30]. PIPYN’s evaluability was limited, as perceived concerns about collection of anthropometric measures (particularly child BMI for the FBI) resulted in incomplete data, preventing assessment of intervention impacts on overweight and obesity within the study timeframe. Stakeholders, particularly local practitioners, reported that existing tools (e.g., long forms, weight measures) felt inappropriate for their FBI offer, reinforcing the need for streamlined, context‑sensitive monitoring and evaluation frameworks built into programmes from the outset.

Evaluating complex system interventions is inherently challenging due to the adaptive and context‑specific nature of the systems within which they operate [28, 50]. Local teams are often expected to deliver programmes while simultaneously evaluating and monitoring outcomes, creating tensions in programme delivery [52, 53]. Frameworks like ENCOMPASS [38] provide best practice guidance and emphasise the importance of iterative evaluation cycles to capture system dynamics over time. However, implementing these fully iterative cycles can be difficult within short‑term, precariously funded community programmes, where workforce capacity, data infrastructure and continuity are constrained. As such, there is a need for evaluation approaches that retain the core principles of systems‑oriented, iterative learning, while also being practical, flexible and proportionate enough to be embedded from the outset and delivered “from the ground up” in real‑world settings. Developing robust yet feasible evaluation frameworks for overweight‑ and obesity‑related WSAs is therefore essential, and tools that enable practitioners to assess multilevel impacts, monitor equity, and align with national guidance on tracking outcomes such as zBMI are needed [43].

At minimum, publicly funded childhood obesity programmes require routine monitoring of theoretically aligned intermediate outcomes (e.g., diet, physical activity, family practices), as these are crucial where collection of anthropometric data is not feasible. Without capturing such outcomes, learning is constrained, accountability is weakened, and the case for sustaining or scaling effective WSAs is undermined [54]. There also remains a need to continuously evaluate outcomes overtime to assess whether programmes risk becoming inequitable over time, for example, using the PROGRESS framework (place, race/ethnicity, occupation, gender, religion, education, socioeconomic status, social capital) [54]. Furthermore, although unintended consequences were explored through the documentary review and stakeholder interviews, available evidence was insufficient to determine if such effects were present, likely reflecting the early stage of implementation, limited routine monitoring data, and the absence of explicit indicators designed to capture unintended impacts. Clear system boundaries and practitioner‑friendly monitoring tools are essential to avoid under‑evaluating programmes with genuine potential for impact.

### Strengths and limitations

This study offers insight into implementation of a WSA approach with an embedded community-based weight management intervention across three different contexts. Combining interviews with a documentary review allowed for the exploration of programme evolution from its inception to the later stages of delivery, and captured stakeholder perceptions of impact, facilitators and barriers at both national and local level. Future evaluations should aim to explore the participating families’ experiences of FBI sessions and their local food and physical activity environments to capture the direct influence of the FBI on participating families and of the wider PIPYN programme on local communities.[55, 56].

A strength of this evaluation was that it was conducted by an independent but embedded research team within Public Health Wales, the organisation responsible for implementing the PIPYN programme. This positioning enabled the research team to co‑develop research methods with stakeholders involved in programme design and draw on established relationships with local delivery teams. In public health settings, this approach has been promoted to build trust and enable capacity for research and evaluation [57]. While this approach carries a potential risk of bias, diverse and often contrasting perspectives were captured across stakeholder interviews, including critical reflections on programme components. Nonetheless, future research should continue to consider who conducts evaluations, and how evaluation structures can be designed to maintain rigour. This is particularly important given the increasing shift toward programme and practitioner teams taking responsibility for designing and conducting their own evaluations within community settings [53].

## Conclusion

This study highlights a need for well-defined intervention boundaries and clear specification of the systems being targeted to guide the set-up and implementation of WSAs. A major priority for the future is the development and promotion of applied, practice-oriented guidance for implementing WSAs. Monitoring and evaluation metrics that are clear and practice-friendly are needed from the outset of programme delivery. This study highlights the critical role of WSAs in bringing together organisations and services that promote a healthy weight agenda within communities. Embedding a locally tailored, community-based family intervention within a WSA can help address systemic inequalities, particularly in areas facing the highest burden of childhood obesity. In turn, strong cross sector partnerships, flexible community informed delivery models, and feasible outcomes-focussed evaluation frameworks are central to understanding, monitoring and sustaining effective implementation. Future research should include family perspectives and explore how early guidance, delivery settings and evaluation approaches influence engagement, equity, and long-term system and population outcomes.

## Supplementary Information

Supplementary Material 1. Perceived mechanisms of change or impact across the pilot areas. Supplementary Material 2. Pre-existing system and Implementation context across the pilot areas. Supplementary Material 3. Adaptations to area specific context across the pilot areas. Supplementary Material 4 .Theoretical framework and interview guide.

## Data Availability

The data underlying this article will be shared on reasonable request to the corresponding author.

## Abbreviations

BMI: Body mass index
CFP: Children and Families Pilot
COM-B: Capability, Opportunity, Motivation–Behaviour (model)
CoP: Community of Practice
CPD: Continuing Professional Development
FBI: Family-Based Intervention
FSW: Family Support Worker
LA: Local authority
NICE: National Institute for Health and Care Excellence
PIPYN: Pwysau Iach Plant Yng Nghymru (Healthy Children Healthy Weight in Wales)
WSA/WSAs: Whole System Approach/ Whole System Approaches
WSAHW: Whole Systems Approach to Healthy Weight programme
zBMI: Body mass index z-score
