# Supplementary material for: Implementation evaluation of a whole systems approach (WSA) to childhood overweight and obesity in local communities: findings from the Children and Families Pilot in Wales, UK
Source: BMC Public Health. 2026 May 4;26:1946. doi: 10.1186/s12889-026-27489-9 (PMC13289350; doi:10.1186/s12889-026-27489-9)
Supplement: Supplementary file 1 — Supplementary Material 1. Perceived mechanisms of change or impact across the pilot areas. Supplementary Material 2. Pre-existing system and Implementation context across the pilot areas. Supplementary Material 3. Adaptations to area specific context across the pilot areas. Supplementary Material 4 .Theoretical framework and interview guide. [file 12889_2026_27489_MOESM1_ESM.docx]

**Supplementary Material 1**

Perceived mechanisms of change or impact across the pilot areas.

|  | Pilot Area A | Pilot Area B | Pilot area C |
| --- | --- | --- | --- |
| System level actions, including relationship building | Key partners included health visiting teams, school nursing, leisure and third sector and Flying Start.  As the project unfolded, there were signs of system approach developing, with a focus on strengthening the leadership, improving knowledge of systems language and tools.  Collaborations with stakeholders and local population through community engagement events and meetings led to growth in the local networks. Improving access to food banks, supporting food and nutrition education and school food provision, creating opportunities for active travel and use of sport facilities, and reaching underserved groups and populations (e.g., those living in social housing and families in social care) are some of the examples of what was achieved as a result. | Key partners included local public health team, health visiting, school nursing, GPs, Health and Wellbeing Promoting Schools Practitioners, oral health improvement initiative, Sports organisations, Flying Start, Families First and other local health initiative partners.  A multi-disciplinary reference group was established to support engagement and co-development with ethnic minority groups. | Key partners included health visiting, school nursing services, midwifery, GPs, Public service boards, Health and Wellbeing Promoting Schools Practitioners, leisure services, Local education department, National Exercise Referral scheme (NERs) and Food Poverty Alliance.  Coordinated by the public health dietetics team, prioritised strategic planning, workforce readiness, service accessibility, and awareness of local nutrition and physical activity initiatives. |

**Supplementary Material 2**

Pre-existing system and Implementation context across the pilot areas.

|  | Pilot Area A | Pilot Area B | Pilot area C |
| --- | --- | --- | --- |
| Pre-existing system and Implementation context | An area characterised by its rurality and pockets of higher deprivation areas; they had no weight management offer for children and young people prior to PIPYN.  A new project team was established in the local health board to deliver PIPYN, alongside a partnership group that brought together community-level stakeholders. | An urban region with ethnically diverse communities with increased risk of overweight and obesity.  The area already had a developing whole systems approach around healthy weight, with a number of established initiatives relating to nutrition and physical activity, including a dietetics-led early years family-based intervention for families with 5-year-old children, as well as a dedicated weight management service for children and young people.  There was an identified gap in services reaching socially disadvantaged areas, including ethnic minority groups. | An area with high deprivation levels and childhood overweight and obesity rates.  Prior to PIPYN, the area had a well-established childhood obesity steering group with a focus on whole systems approaches for early prevention of overweight.  The area also had experience of a previous pilot for a family-based programme aimed at parents and carers of children aged 0 – 5 years old. However, it was not seen as successful, resulting in an ongoing gap in service provision with no dedicated child weight management programme at the time. |

**Supplementary Material 3**

Adaptations to area specific context across the pilot areas.

|  | Pilot Area A | Pilot Area B | Pilot area C |
| --- | --- | --- | --- |
| Adaptations to the area specific context | Further priority areas for action were identified following system definition and causal mapping exercises: active travel, access to healthy food, and promotion of healthy rewards for children.  The FBI offer was developed in line with Family Support Worker Resource Pack and Manual, as a one-to-one home-based intervention with topic-focused sessions guided by the family’s needs and delivered following a referral from a Health Visitor. The eligibility criteria were as defined in the PIPYN programme outline. However, the offer was met with lack of referrals and uptake among the families, citing the referral criteria and families not accepting home-based support as a reason. The offer was later adapted to include school-based delivery and group sessions, supported by a stronger social media presence to increase the profile of FBI among the community. | The FBI was offered as a part of the existing array of programmes already available within the local health board.  Referrals to the FBI were initially primarily from health visitors, with self-referrals also accepted. A dietetic assistant practitioner, supported by a dietitian, delivered the intervention. Following an early pilot and reference group consultation, a structured programme was developed, offering families a choice of group or one-on-one sessions.  Accessibility barriers, particularly concerning language diversity and reading proficiency, were identified in reports, especially regarding translation of programme materials and questionnaires. To address this, further co-development of materials was planned, including the creation of better tailor resources to the target population. Referral approaches also expanded through developing local networks, incorporating non-clinical routes like social media outreach and leveraging the connections of local family support workers to build rapport and trust. | Delivery focused on collaboratively embedding and co-developing interventions with local partners and families, deviating from national eligibility and delivery models.  The area adopted an open recruitment in high-obesity catchment areas, offering a mix of group-based, face-to-face, and online activities, including a family-based education programme and optional one-on-one support (though uptake was low).  Strong family engagement, particularly high self-referrals, led to the FBI recognised as a "fully inclusive, universally accessible offer." There was lack clarity around targeted aspects of FBI, including if families attending met national eligibility criteria. |

**Supplementary Material 4**

Theoretical framework and interview guide.

| **Framing** | **Example questions** |
| --- | --- |
| **Introduction** | - Can you tell us a little bit about your career history to date, and then your current involvement with Children and Families Programme (CFP) also known as PIPYN? - What do you think the aims of PIPYN are? - What would success for PIPYN look like for you? |
| **Implementation:** What is implemented, and how? | - To what extent, if at all, have you used systems thinking and/or approaches when implementing PIPYN? - What, if anything has changed or has started to change since this work has began? - What are the barriers and facilitators to implementing PIPYN? - In relation to the FBI specifically, how did you go about reaching your target population? |
| **Mechanisms of impact:** how does the delivered intervention produce change? | - The programme is intended to influence healthy behaviours, including a) food and nutrition and b) physical activity. In your view, to what extent has PIPYN supported this so far? - To what extent have early years settings (such as schools, nurseries and play groups) and local organisations been involved in PIPYN so far? |
| **Context:** how does context affect implementation and outcomes? | - For the programme in your area specifically, what broader factors may promote the success of, or inhibit, PIPYN? - Were there sectors or organisations that you felt were harder to reach by PIPYN, or less likely to engage? |
| **Unintended impact or consequences:** what are the ripple effects of the intervention? | - Can you tell us about any other unexpected impacts of PIPYN in your programme area? These can be positive or negative. |
